# Descriptive Analysis and Factors Associated With Relapse in Dogs With Presumptive Idiopathic Immune‐Mediated Polyarthritis

**DOI:** 10.1111/jvim.70241

**Published:** 2025-09-05

**Authors:** Diane Pichard, Thibaud Robin, Kevin Le Boedec, Christelle Maurey, Maxime Kurtz, Fiona Da Riz, Stéphane Blot, Morgane Canonne‐Guibert, Mario Cervone, Emilie Krafft, Jean‐Luc Cadore, Loïc Desquilbet, Ghita Benchekroun

**Affiliations:** ^1^ Ecole Nationale Veterinaire d'Alfort—CHUV‐AC Maisons‐Alfort France; ^2^ Centre Hospitalier Veterinaire Fregis—IVC Evidensia France, Service de Médecine Interne Paris France; ^3^ Ecole Nationale Veterinaire d'Alfort—CHUV‐AC—IMRB Maisons‐Alfort France; ^4^ Clinique Vétérinaire Evolia L'Isle‐Adam France; ^5^ Département des Animaux de Compagnie, de Loisir et de Sport, Service Clinique de Médecine Université de Lyon, VetAgro Sup, Campus Vétérinaire de Lyon Marcy l'Etoile France; ^6^ Ecole Nationale Veterinaire d'Alfort—IMRB Maisons‐Alfort France

**Keywords:** canine, CRP, IMPA, outcome, predictive factors, remission

## Abstract

**Background:**

Immune‐mediated polyarthritis (IMPA) is a joint disease common in dogs. Although its prognosis is generally favorable, relapses are frequent, and predictive factors for relapse remain poorly characterized.

**Objectives:**

To evaluate the outcome of medical management of IMPA in dogs and identify predictive factors for relapse.

**Animals:**

Client‐owned dogs diagnosed with non‐associative IMPA between 2010 and 2022 across four veterinary referral centers.

**Methods:**

This was a retrospective multicentric study. The data collected at the time of diagnosis, including signalment, clinical presentation, imaging, and laboratory findings, and treatments, were analyzed. Relapse was defined as the recurrence of clinical signs, CRP levels above the reference range, or both after remission. Associations with time from remission to relapse were assessed using univariate and multivariable Cox models.

**Results:**

Among 119 dogs, 114 (95.8%) achieved remission. Among the 85 dogs with relapse data, the median time to relapse was 6.5 months, with relapse rates of 43% at 6 months, 61% at 12 months, and 65% at 24 months. In the multivariable analysis, thrombocytosis (adjusted hazard ratio [aHR] = 5.5 [2.0–15.0]_95%_, *p* < 0.001), lymphadenomegaly (aHR = 4.0 [1.5–11]_95%_, *p* = 0.006) and lameness (aHR = 3.9 [1.2–12.2]_95%_, *p* = 0.02) at initial admission were independently and significantly associated with time from remission to relapse.

**Conclusion:**

This study highlights a favorable clinical outcome for dogs with non‐associative IMPA with high remission rates but substantial relapse risks. Lameness, lymphadenomegaly, and thrombocytosis are associated with a greater risk of relapse.

**Abbreviations**
ACVIM: American College of Veterinary Internal Medicine
ALP: alkaline phosphatase
BPM: beats per minute
CRP: C‐reactive protein
CT: computed tomography
IMPA: immune‐mediated polyarthritis
MPM: movements per minute
PUPD: polyuria‐polydipsia
SIRS: systemic inflammatory response syndrome
SRMA: steroid‐responsive meningitis and arteritis
USG: urine specific gravity

## 1 Introduction

Immune‐mediated polyarthritis (IMPA) is a common inflammatory joint disease in dogs that is characterized by inflammation in multiple joints. IMPA can be classified as non‐associative (primary) or associative (secondary), with the latter triggered by exogenous factors (e.g., vaccination, drugs) or endogenous extra‐articular disorders (e.g., infections, neoplasia) [1, 2, 3].

The goal of therapy is remission with minimal medication to prevent relapses. Treatment involves immunosuppressive drugs, with corticosteroids as first‐line therapy [1, 4, 5, 6]. Other agents, including ciclosporin, mycophenolate mofetil, leflunomide, and azathioprine, are also used [4, 5, 6, 7, 8]. No consensus exists on the efficacy of monotherapy versus combination therapy [2]. In large breeds, adding a second immunosuppressant early is recommended for faster steroid‐sparing effects [1, 9]. Studies report that prednisolone alone is as effective as combination therapy [1, 10, 11], with similar efficacy noted between prednisolone and ciclosporin [7]. However, adding leflunomide to corticosteroids has shown no significant benefit in several studies [6, 12, 13].

Although the prognosis is generally good, relapse rates are high. Retrospective studies report variable remission rates ranging from 56% to 95%, and relapse rates vary from 20% to 53%. Relapses often occur within the first year [1, 3, 10, 14, 15]. Few objective biomarkers are currently available to quantify remission or detect relapse in dogs with non‐associative IMPA. Serum C‐reactive protein (CRP) levels are frequently used to monitor immune‐mediated diseases [16]. One study reported elevated serum CRP levels in all cases of non‐associative IMPA, which decreased rapidly with treatment, supporting its use in treatment monitoring [17]. However, the serum CRP sensitivity for detecting poorly controlled cases is low (13%) [18]. The serum CRP‐to‐albumin ratio is a potential disease activity marker in human rheumatoid arthritis patients [19, 20, 21]. In dogs, the predictive factors of relapse remain poorly characterized. The objectives of the study were to describe the outcomes of dogs with non‐associative IMPA medically managed and to identify predictive factors for relapse.

## 2 Materials and Methods

### 2.1 Study Design

**FIGURE 1:**
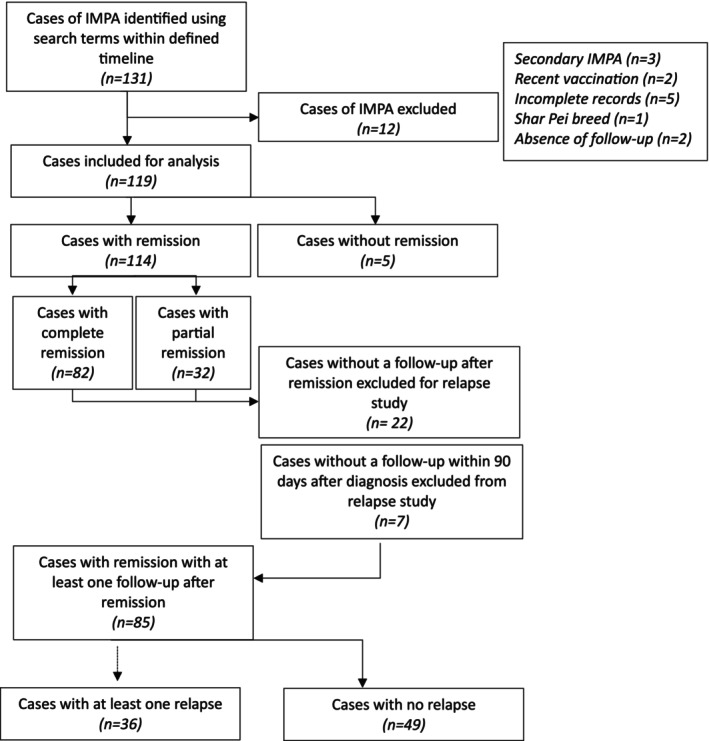
Flow chart of group characterization for remission and relapse studies.

A retrospective multicentric study was designed. Dogs that were diagnosed with non‐associative IMPA and presented to Alfort Teaching Veterinary Hospital, Fregis Veterinary Hospital Center, VetAgroSup Teaching Veterinary Hospital, and Evolia's veterinary clinic between 2010 and 2022 were included. The cases were included by searching clinical data servers using the following keywords: polyarthritis, dog, joint aspiration, and synovial fluid cytology. The inclusion criteria were diagnosis of non‐associative IMPA and at least one follow‐up examination, 7 days after diagnosis. For remission analysis, an available CRP value was an inclusion criterion. For relapse analysis, the inclusion criteria was at least one follow‐up, with a minimum of 7 days after remission. This threshold of 7 days was fixed to ensure a minimum duration of follow‐up. Diagnosis of non‐associative IMPA was minimally based on consistent clinical signs (lameness, reluctance to walk, pyrexia, hyporexia or anorexia, weight loss, and joint swelling), neutrophilic inflammation reported in at least two joints at cytological examination of the joint tap, and exclusion of an underlying disorder (using complete hematologic, biochemical and urinalysis workup; thoracic and abdominal imaging [abdominal and thoracic CT scans or abdominal ultrasound and thoracic radiographs], serological or PCR evaluation for infectious diseases; and absence of vaccine or drug administration within the month before clinical signs occurred). Erosive and non‐erosive forms of polyarthritis were differentiated on the basis of joint radiographs or computed tomography; both forms were included. Dogs treated with immunomodulatory drugs (prednisolone or other immuno‐suppressive agents) within the 2 weeks prior to diagnosis, dogs with specific breed‐associated IMPA (Shar Pei, Akita or Greyhound) or incomplete medical records were excluded (Figure 1).

Partial remission was defined as improvement without complete resolution of clinical signs, a persistently high serum CRP concentration (decrease of less than 50% of the initial value) or both. Complete remission was defined as entire resolution of clinical signs and a serum CRP concentration within the reference range. Cases were classified according to retrospective clinical record information.

Relapse was defined as recurrence of clinical signs, an increase in the serum CRP concentration without any other identified cause, or both after remission (complete or partial). Dogs without a follow‐up within 90 days postdiagnosis were excluded because of potential inaccuracies in determining their remission date.

### 2.2 Data Collection

Data extracted from the medical records at diagnosis included breed, sex, neuter status, age, weight, size (0–10 kg; > 10–20 kg; > 20–40 kg; > 40 kg), presence or absence of the following clinical signs (pyrexia [defined as rectal temperature > 39.5°C]), inappetence, anorexia, weight loss, gastrointestinal clinical signs (vomiting, diarrhea), peripheral lymphadenomegaly, presence of signs usually used as systemic inflammatory response syndrome (SIRS) criteria (at least two criteria from: heart rate > 120 beats/min for large breed dogs and > 140–160 beats/min for small and medium breeds, respiratory rate > 20 breaths/min, rectal temperature > 39.2°C or < 38.1°C, neutrophil concentration < 6000/mm^3^ or > 16 000/mm^3^ or band cells > 3%), lameness, reluctance to move, cervical hyperesthesia, painful, warm or swollen joints, synovial distension, and ligament laxity. For all dogs, when available, biological results were recorded (CBC, serum biochemistry, urinalysis, serum CRP concentration, urine culture results, urine protein–creatinine ratio, serological or blood PCR analysis for vector‐borne diseases [ *Anaplasma phagocytophilum* , *Anaplasma platys* , *Ehrlichia canis* , *Ehrlichia ewingii* , *Ehrlichia chaffeensis* , *Borrelia burgdorferi* , *Dirofilaria immitis* , *Leishmania infantum* ]) and blood culture results. Joint fluid analysis (cytological examination and, when available, bacterial culture), cerebrospinal fluid analysis when available (cytology, protein concentration), number of joints sampled, number of affected joints, joint ratio (proportion of number of affected joints among number of sampled joints), erosive status, and initial treatment (defined as therapeutic protocol started or maintained during the first 7 days following diagnosis, [drug, dose, duration, monotherapy or bitherapy]) were also recorded. At each follow‐up, the time from diagnosis, clinical history from the previous visit, clinical examination, clinicopathological data (CBC, biochemistry, serum CRP concentration) and treatment modifications were extracted. The occurrence of remission, time to reach remission, potential relapse, and number of relapses were recorded.

### 2.3 Statistical Analysis

Continuous variables are presented as medians and interquartile ranges (1st quartile–3rd quartile). Descriptive categorical variables are reported as frequencies and percentages (%). To identify exposures associated with relapse occurrence, survival analyses were conducted using the Kaplan–Meier method and univariate and multivariable Cox proportional hazard models. Univariate and multivariable analyses were only conducted for relapse events because of suspected imprecise or poorly estimated remission dates, which are likely overestimated for some animals. This difference is because remission was not a reason for veterinary visits after IMPA diagnosis, and some owners might have delayed the follow‐up visits required to confirm remission.

For the remission survival analysis, the starting point was the date of IMPA diagnosis, the event date was the date of remission, and the censoring date was the date of all‐cause death or of the last follow‐up recorded during disease monitoring. For the relapse survival analysis, the starting point was the date of remission, the event date was the first relapse, and the censoring date was the date of all‐cause death or of the last follow‐up recorded during disease monitoring. Dogs were excluded from the relapse survival analysis if they did not have one follow‐up after remission and if they did not have a confirmed remission date within 90 days after diagnosis. This 90‐day duration was selected to ensure sufficient clinical documentation for reliable timing of remission status in the context of a retrospective study.

Continuous exposures were converted into binary exposures on the basis of a relevant clinical threshold or previous studies [22, 23, 24, 25]. In particular, the thresholds for the serum CRP concentration and the serum CRP/albumin ratio were determined on the basis of current literature [16, 17, 19]. The following exposures were used as binary factors: sex, neutered status, Bernese mountain breed, age (older than 5 years), erosive status, weight (over 20 kg), lethargy, lameness, cervical pain, hyporexia, SIRS status, presence of gastrointestinal signs (diarrhea, vomiting, regurgitation), hyperthermia (≥ 39.5°C), weight loss, inflammation detected in more than 75% of the joints sampled, neutrophilic leukocytosis (leukocyte concentration > 17 000/mm^3^ and neutrophil concentration > 10 000/mm^3^), serum CRP concentration higher than 50 mg/L and serum CRP/albumin ratio higher than 3, anemia (hematocrit < 35%), thrombocytosis (> 500 000/μL), azotemia (serum creatinine > 14 mg/L), type of treatment (single therapy with corticosteroids or corticosteroids combined with another immunosuppressive drug) and corticosteroid dosage initiated at diagnosis or within 7 days after diagnosis at a dose higher than 1.5 mg/kg. The type of remission (complete or partial) was used as an exposure for relapse survival analysis. The exposures included in the stepwise selection process were those with a *p* value < 0.20 in the univariate analysis for relapse survival analysis, whereas those with missing data for more than 25% of the dogs or an insufficient sample size (< 5 dogs exposed) were excluded. Variables retained from this stepwise procedure were incorporated into the multivariable Cox proportional hazards model. In the stepwise selection process, the *p* value for entry was set at 0.157, and the *p* value for removal was set at 0.157 [22, 23]. The hazard assumption was visually checked by inspection of Kaplan–Meier curves. The significance threshold was set at 0.05. All the statistical analyses were performed using commercially available software (SAS University Edition).

## 3 Results

### 3.1 Animals

A total of 131 dogs diagnosed with non‐associative IMPA from 2 teaching veterinary hospitals and 2 private practice hospitals were included in the study. Twelve dogs were excluded because of a diagnosis of an underlying cause (*n* = 3), recent vaccination (*n* = 2), incomplete medical records (*n* = 5), breed (Shar Pei; *n* = 1) or no follow‐up available (*n* = 2), leading to the inclusion of 119 dogs. There were 112 purebred dogs and 7 crossbreeds. The most common purebred dogs included (> 5 dogs) were Bernese Mountain dogs (*n* = 15), Labrador dogs (*n* = 11), and Shih‐Tzu dogs (*n* = 6). Forty‐five breeds were recorded with ≤ 5 dogs. The median age and weight were 4.8 years [3.0–7.0] and 15.6 kg [6.7–29.5], respectively. Female dogs (neutered (*n* = 42), intact (*n* = 26)) and male dogs (neutered (*n* = 14), intact (*n* = 37)) represented 57.1% (68/119) and 42.9% (51/119) of the dogs, respectively.

### 3.2 Clinical, Clinicopathological, and Imaging Findings and Treatments

Overall, the most common complaints at presentation were lethargy (78.2%, 93/119), reluctance to walk (76.5%, 91/119), lameness (67.2%, 80/119), hyporexia (52.1%, 62/119), weight loss (24.4%, 29/119) and gastrointestinal signs (22.7%, 27/119). Clinical examination revealed joint swelling in more than two joints (61.3%, 73/119), a rectal temperature ≥ 39.5°C (60.5%, 69/114), peripheral lymphadenomegaly (30.3%, 36/119) and cervical pain (17.7%, 21/119). Among dogs with cervical pain, 10 had cerebrospinal fluid analysis performed (48%, 10/21), and only one dog showed neutrophilic inflammation. Systemic inflammatory response syndrome was identified in 63.3% of the dogs (74/117). The median serum CRP concentration was 95.1 mg/L [29.3–167.3], and the serum CRP concentration was greater than 50 mg/L in 60/96 dogs (63%). The median serum CRP/albumin ratio was 3.0 [1.2–5.5]. The serum CRP/albumin ratio was greater than 3 in 50% (39/78) of the dogs. Azotemia, anemia, thrombocytosis, and neutrophilic leukocytosis were detected in 5/89 (6%), 5/102 (4.9%), 13/99 (13%) and 52/101 (51.5%) dogs, respectively. The most common polyarthritis type identified in this cohort was non‐erosive polyarthritis (92.8% 103/111). Among the 86 dogs for which data were available, the median number of joints sampled was 4.0 [3.0–5.0], and the joint ratio was > 75% in 80% (68/85) of the cases. Corticosteroids were used as the sole therapy in 76.3% of the dogs (87/114). An additional immunosuppressive agent was added within the first week post diagnosis in 27/114 dogs (23.7%). Additional immunosuppressive agents used included azathioprine in 44% of the dogs (12/27), mycophenolate mofetil in 30% of the dogs (8/27), ciclosporin in 22% of the dogs (6/27) and leflunomide in 4% of the dogs (1/27). At the time of discharge, the median prednisolone dose was 1.8 mg/kg [1.2–2.0], with 72 out of 110 dogs (65.5%) receiving a dosage above 1.5 mg/kg. The first prednisolone dose reduction was performed in 73 of 114 dogs, occurring at a median of 20 days [14.5–32.5] after treatment initiation. Among the 60 dogs for whom data were available, the median dose decrease at first reduction was 49% [27–59], with a resulting median prednisolone dose of 1.0 mg/kg [0.5–1.2]. Relapse occurred in 36 dogs, of whom only 2 (6%) were off all medications at the time of relapse; 6 of the 34 relapsing dogs for which data were available (18%) were receiving two immunosuppressive agents. The demographic data, clinical signs, clinicopathological results, and treatment characteristics of the dogs included are summarized in Table 1.

**TABLE 1:** Demographic data, clinical signs, laboratory findings, and treatment characteristics in all dogs with immune‐mediated polyarthritis included.

Variables	Remission (*n* = 119)
Signalment	
Median age (years)	4.8 [3.0–7.0]
Female: male	68:51
Neutered: intact	56:63
Bernese Mountain Dog	15/119 (12.6%)
Median weight (kg)	15.6 [6.7–29.5]
Weight ≥ 20 kg	52/119 (43.7%)
Size	
0–10 kg	25/119 (21.0%)
> 10–20 kg	42/119 (35.3%)
> 20–40 kg	40/119 (33.6%)
> 40 kg	12/119 (10.1%)
Clinical signs	
Lethargy	93/119 (78.2%)
Reluctance to walk	91/119 (76.5%)
Lameness	80/119 (67.2%)
SIRS	74/117 (63.3%)
Joint swelling	73/119 (61.3%)
Hyperthermia (≥ 39.5°C)	69/114 (60.5%)
Hyporexia	62/119 (52.1%)
Lymphadenomegaly	36/119 (30.3%)
Weight loss	29/119 (24.4%)
Digestive signs	27/119 (22.7%)
Cervical pain	21/119 (17.7%)
Laboratory results	
CRP ≥ 50 mg/L	60/96 (62.5%)
Median [range] CRP (mg/L)	95.1 [29.3–167.3]
Neutrophilic leukocytosis	52/101 (51.5%)
CRP/Alb ratio ≥ 3	39/78 (50.0%)
Median [range] CRP/Alb ratio	3.0 [1.2–5.5]
Thrombocytosis	13/99 (13.1%)
Azotemia	5/89 (5.6%)
Anemia	5/102 (4.9%)
Treatment	
Corticosteroids alone	87/114 (76.3%)
Addition of a second immunosuppressive agent	27/114 (23.7%)
Azathioprine	12/27 (44.4%)
Mycophenolate mofetil	8/27 (29.6%)
Ciclosporin	6/27 (22.2%)
Leflunomide	1/27 (3.7%)

Abbreviations: Alb, Albumin; CRP, C‐reactive protein; SIRS, systemic inflammatory response syndrome.

### 3.3 Remission

**FIGURE 2:**
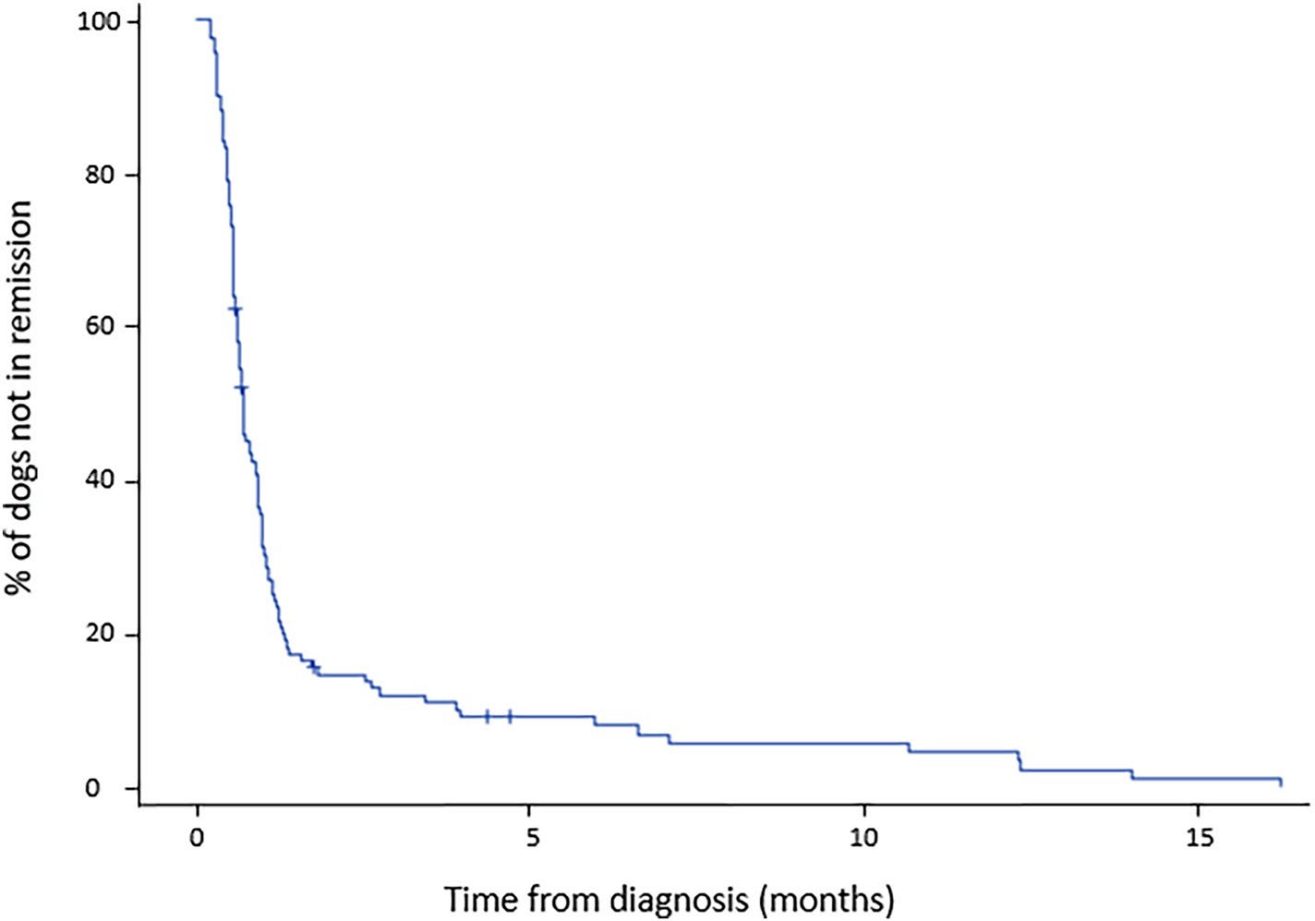
Kaplan–Meier estimates of remission rates from IMPA diagnosis. « + » signs indicate the censored case.

One hundred and fourteen dogs (95.8%, 114/119) achieved partial (28.1%, 32/114) or complete (71.9%, 82/114) remission. All partial remissions were based on both persistent CRP elevation and ongoing clinical signs or abnormal physical examination findings. The median time from diagnosis to the first follow‐up showing remission (as estimated by the Kaplan–Meier method) was 22 days [16–36]. At 3 and 6 months after diagnosis, the estimated remission rates for all remissions were 88.1% [82.1–94.0]_95%_ and 92.0% [86.9–97.1]_95%_, respectively (Figure 2). All cases of erosive IMPA (7.6%; 8/106) achieved remission (4/31 (13%) achieved partial remission, 4/75 (5%) achieved complete remission).

### 3.4 Relapse

Among the 114 dogs that achieved remission, the date of remission recorded was not within 90 days after diagnosis in 7 dogs, and a follow‐up after remission was not available for 22 dogs. The median total follow‐up duration for those 85 dogs included in the relapse analysis was 121 days [53–211]. For dogs who experienced a relapse, the median total follow‐up was 123 days [55–231], whereas for those who did not relapse, it was 119 days [48–177]. For all 85 dogs with sufficient follow‐up data to be included in the relapse analysis, Kaplan–Meier and Cox analyses were performed. Among these dogs, 49 dogs were censored because of the absence of relapse during follow‐up after remission (48/49 dogs were censored at the date of last follow‐up and 1 dog was euthanized at 82 days after remission), and 36 dogs experienced at least one relapse. This included 29 dogs, 5 dogs, 1 dog, and 1 dog that experienced 1, 2, 3, and 4 relapses, respectively. Among the 85 dogs, 3 had an erosive form (4% 3/85), and 2/3 presented at least 1 relapse.

**FIGURE 3:**
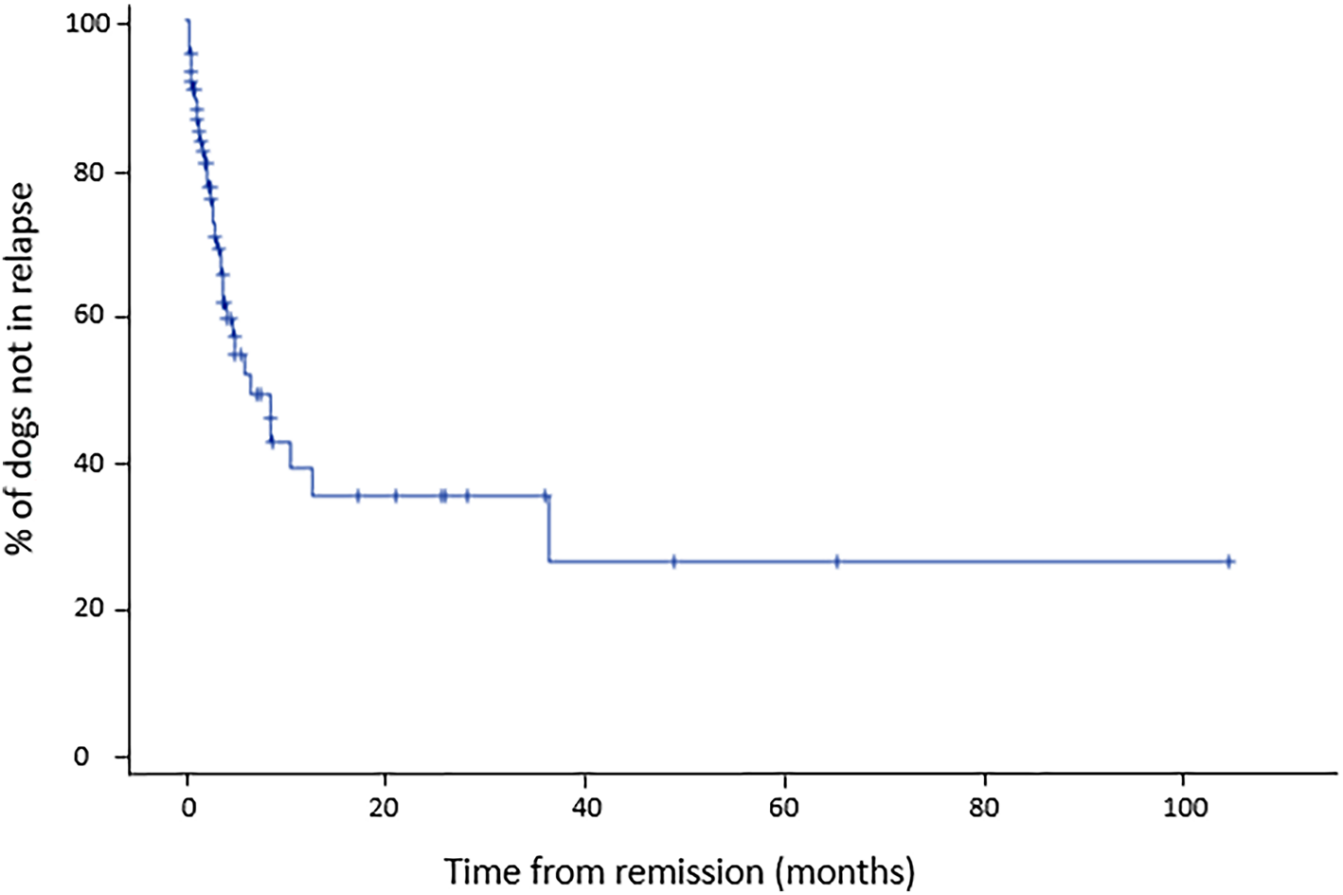
Kaplan–Meier estimates of the relapse rate from IMPA diagnosis. « + » signs indicate the censored case.

The median time from remission to first relapse was estimated to be 6.5 months [2.8–10.6] using the Kaplan–Meier method. The estimated relapse rates were 43% [31–55]_95%_ at 6 months (24 dogs at risk), 61% [48–75]_95%_ at 12 months (11 dogs at risk) and 65% [50–79]_95%_ at 24 months (7 dogs at risk; Figure 3).

On univariate analysis, age ≥ 5 years (hazard ratio [HR] = 2.8 [1.4–5.7]_95%_, *p* < 0.01), azotemia at admission (HR = 3.6 [1.0–12.0]_95%_, *p* = 0.04) and thrombocytosis at admission (HR = 3.4 [1.5–7.7.7]_95%_, *p* = 0.004) were significantly associated with relapse occurrence (Table 2). Age ≥ 5 years, being neutered, lameness, peripheral lymphadenomegaly, hyporexia, gastrointestinal signs, type of remission, azotemia, neutrophilic leukocytosis, and thrombocytosis were included as candidate variables in the stepwise selection process. The final multivariable Cox model included animals with azotemia, thrombocytosis, peripheral lymphadenomegaly, and lameness at admission. Independently of azotemia, thrombocytosis (adjusted HR [aHR] = 5.5 [2.0–14.7]_95%_, *p* < 0.001), peripheral lymphadenomegaly (aHR = 4.0 [1.5–11.0]_95%_, *p* = 0.006), and lameness (aHR = 3.9 [1.2–12.2]_95%_, *p* = 0.02) were significantly associated with the time to relapse occurrence.

**TABLE 2:** Results of univariate and multivariable Cox analysis for time to IMPA relapse.

Exposure variables	Number of dogs (*n* = 85)	Univariate analysis		Multivariable analysis	
		HR [95% CI]	*p*	HR [95% CI]	*p*
Age ≥ 5 years old	43/85 (51%)	2.8 [1.4–5.7]	**0.005**	^b^	
Thrombocytosis	10/69 (15%)	3.4 [1.5–7.7]	**0.004**	5.5 [2.0–14.8]	**< 0.001**
Azotemia (serum creatinine > 14 mg/L)	5/64 (8%)	3.6 [1.0–12.0]	**0.04**	3.1 [0.7–14.0]	0.15
Hyporexia	48/85 (56%)	1.9 [0.9–3.8]	0.07	^b^	
CRP/Alb ratio ≥ 3^a^	29/56 (52%)	2.2 [0.9–5.3]	0.07		
Lymphadenomegaly	25/85 (29%)	1.7 [0.9–3.4]	0.10	4.0 [1.5–10.9]	**0.006**
Anemia^a^	2/71 (3%)	5.8 [0.7–47.0]	0.10		
Type of remission (complete)	62/85 (73%)	0.6 [0.3–1.2]	0.12	^b^	
Neutered status (entire)	44/85 (52%)	1.6 [0.8–3.1]	0.16	^b^	
Neutrophilic leukocytosis	39/71 (55%)	1.7 [0.8–3.6]	0.17	^b^	
Lameness	57/85 (67%)	1.7 [0.8–3.5]	0.19	3.9 [1.2–12.2]	**0.02**
Digestive signs	23/85 (27%)	1.6 [0.8–3.2]	0.19	^b^	
Cervical pain	14/85 (17%)	1.5 [0.7–3.5]	0.33		
Treatment (combined)	21/82 (26%)	0.7 [0.3–1.6]	0.37		
CRP ≥ 50 mg/L	43/67 (64%)	1.4 [0.6–2.9]	0.41		
SIRS	56/84 (67%)	1.4 [0.6–2.9]	0.43		
Joint ratio ≥ 75%	51/60 (85%)	2.5 [0.2–35.0]	0.50		
Temperature ≥ 39.5°C	49/81 (61%)	1.2 [0.6–2.4]	0.62		
Lethargy	67/85 (79%)	0.8 [0.4–1.8]	0.64		
Gender (female)	45/85 (53%)	0.9 [0.4–1.6]	0.66		
Bernese Mountain Dog	10/85 (13%)	1.3 [0.4–3.6]	0.66		
Weight loss	21/85 (25%)	0.8 [0.4–2.0]	0.66		
Hypercalcemia	2/28 (7%)	1.4 [0.2–11.0]	0.73		
Erosive status	4/79 (5%)	1.2 [0.4–4.0]	0.74		
Weight ≥ 20 kg	39/85 (46%)	1.0 [0.5–1.9]	0.98		

Abbreviations: Alb, albumin; CI, confidence interval; CRP, C‐reactive protein; HR, hazard ratio; joint ratio ≥ 75%, inflammation detected in more than 75% of joints sampled; SIRS, systemic inflammatory response syndrome.

^a^ Variables that were excluded from the stepwise model due to too small sample size or too many missing data.

^b^ Candidates variables to the stepwise selection process that were not selected in the final multivariable model.

## 4 Discussion

This study revealed that several exposures were significantly associated with relapse occurrence in dogs diagnosed with non‐associative IMPA. The estimated relapse rate was 42.7% at 6 months, 61.4% at 12 months, and 64.6% at 24 months. According to the multivariate analysis, the presence of lameness, lymphadenomegaly, and thrombocytosis at the time of diagnosis was significantly associated with the time to relapse.

The high remission rate of 92.0% at 6 months identified in this study is similar to that reported in previous studies [2, 10, 15, 26]. This finding correlates with the results of a recent study, with remission occurring 2 to 3 weeks after treatment was implemented (at first recheck) [15]. Notably, owing to the retrospective nature of the study, the remission date was approximated and defined as the first follow‐up date where remission was confirmed. Thus, remission could have occurred earlier than assessed by the follow‐up date in some cases; thus, overestimation of the time to remission could have occurred. In this study, a distinction between partial (improvement without resolution of clinical signs, persistently high serum CRP concentration or both) and complete (entire resolution of clinical signs and normal serum CRP concentration) remission was made, and the type of remission (partial versus complete) was not significantly associated with the time to relapse.

Bernese Mountain Dogs represented 12.6% of the IMPA cases, while they accounted for only 0.78% of all dogs seen in consultation over the four institutions during the study period. Similarly, Labrador Retrievers comprised 9.5% of the IMPA cohort and 4.83% of the hospital's caseload. These findings could suggest a potential breed predisposition for Bernese Mountain Dogs. Those breeds are not reported as predisposed in the current literature and were, on the contrary, underrepresented in recent studies (no Bernese mountain dogs [1, 10, 15], 4.0% [10] and 7.0% [15] were Labrador Retriever dogs), although Clements et al. reported that 15% of dogs in the study were Labradors [1]. The current literature presents inconsistent findings regarding a possible predisposition of size. Studies have reported conflicting patterns of overrepresentation, with some highlighting large breed dogs (> 20 kg) [1, 9], others focusing on middle breed dogs (< 20 kg) [10], and still others focusing on small breed dogs weighing less than 10 kg [4]. The age distribution observed in this study closely aligns with findings from prior studies, with younger to middle‐aged dogs predominantly affected [1, 2, 4, 9, 10, 15].

In our study, the estimated 61.4% relapse rate at 12 months after remission was higher than those previously reported, varying from 20% to 53% [3, 10, 14, 21]. These differences might be due to a slight difference in relapse definition, as relapse in previous studies has been defined exclusively using clinical relapse. Moreover, the time to relapse is likely underestimated in our study because of the inaccuracy of remission dates. To mitigate this limitation, only dogs with a recorded remission date within 90 days of diagnosis were included in the relapse analysis. However, repeated relapses occurred in 19.4% of relapsing dogs in our study, which is lower than the 50% reported by Sparrow et al., though differences in study design and follow‐up protocols might account for this discrepancy [10, 15]. It has been reported that relapses after 1 year are unlikely. Our study supports this observation, as the relapse rates were 61.4% at 12 months and only slightly increased to 64.5% at 24 months, indicating that most relapses occur within the first year after diagnosis. In the multivariable analysis, thrombocytosis, lameness, and peripheral lymphadenomegaly at presentation were independently and significantly associated with the time to relapse occurrence. Lameness and peripheral lymphadenomegaly are two markers of joint involvement. We suspect that lameness at presentation is a sign of severe and advanced ongoing joint inflammation and, as such, could reflect more severe and difficult‐to‐manage conditions. Although lymph nodes have rarely been sampled for confirmation, lymph node enlargement is highly suspected to be secondary to systemic inflammation. In fact, lymph node enlargement in rheumatoid lymphadenopathies is predominantly influenced by local joint activity [27]. Similar to lameness, we hypothesized that lymphadenomegaly indicates an enhanced immune response and more severe disease, increasing the risk of silently uncontrolled disease and subsequent relapse. Indeed, in rheumatoid arthritis, recent research highlights the importance of metabolic regulation in autoimmunity and tissue inflammation, suggesting that metabolic alterations within the lymph node compartment could influence the initiation of autoimmune responses [28]. Although human rheumatoid arthritis and non‐erosive IMPA in dogs are distinct entities with different pathophysiological mechanisms, these findings provide a useful conceptual framework for considering the potential role of lymphoid tissue in early immune dysregulation. Moreover, unexplained lymphadenopathy diagnosed as lymph node reactive hyperplasia might occur before the onset of articular clinical signs in human patients [29]. In more recent studies, cellular composition analysis of inguinal lymph nodes revealed a higher number of CD19+ B cells in early arthritis patients [30, 31]. It is therefore possible that B‐cell persistence in the lymph nodes is favoring the occurrence of a relapse of non‐associative IMPA in dogs.

In our study, elevated platelet counts are associated with a relapse occurrence [32]. The main causes of secondary thrombocytosis are tissue damage, infection, malignancy, and chronic inflammation [33]. In addition, in humans, inflammatory thrombocytosis is related to higher interleukins 6 (IL‐6) levels [33, 34].

This study has several limitations. Owing to the retrospective design of the study, the completeness and consistency of reported clinical and clinicopathological findings in the medical records, treatment plans, monitoring procedures, and tapering regimens varied. Additionally, dogs with erosive forms were not excluded from the analysis in order to reflect the full clinical spectrum of the disease encountered in practice, even though these cases might introduce some heterogeneity. Animal care was provided by internal medicine and neurology specialists, and treatment decisions might have been influenced by individual clinician preferences and animal‐specific factors. Moreover, multicentric involvement added some variability to case management. Another limitation is the lack of standardization in follow‐up visit timing and the definition of remission date as the first follow‐up with remission assessment. As previously mentioned, this might have led to an overestimation of the time to remission. Additionally, a description of remission based on repeated arthrocentesis could have increased confidence in the definitions of remission and relapse. We acknowledged that the diagnosis of non‐associative IMPA might be uncertain due to the retrospective nature of the study. However, we minimized the risk of including cases of secondary polyarthritis by applying strict exclusion criteria (including the exclusion of any identifiable underlying disorder based on a complete hematologic, serologic or PCR testing for infectious diseases, and the absence of recent vaccination or drug administration within 1 month prior to onset of clinical signs). Additionally, cases with missing key data were excluded, and the final inclusion also relied on the clinical judgment of the attending veterinarian. Moreover, the date of remission was used as the starting point for relapse analysis to better reflect the clinical course; however, due to the retrospective nature of the study and potential uncertainty in precisely determining the time of remission, this might have introduced variability in the relapse timing assessment. As well, it was not always possible to distinguish whether persistent clinical signs in dogs classified as being in partial remission were attributable to ongoing IMPA activity or to concurrent chronic joint conditions. Finally, although our study accounted for several confounding variables, some unmeasured confounders are still possible.

In this study, we included variables with a univariate *p* value < 0.20 as candidates in the stepwise selection process to generate the multivariable model to investigate the association with time to relapse. In the stepwise selection process, *p* values for entry and removal were set at 0.157 and not at 0.05 to limit the noninclusion of relevant confounders [22, 23]. We therefore hypothesized that the exposures that were not included in the stepwise process as well as those that were candidates for the stepwise process but that were ultimately not selected did not play a strong confounding role in our study. Taken together, the significant adjusted associations with time to relapse provide some evidence that thrombocytosis, lameness, and peripheral lymphadenomegaly at presentation might increase the risk for relapse in a dog.

In conclusion, remission frequently occurs in dogs with non‐associative IMPA; however, relapses are common, especially within the first year of treatment. Lameness, lymphadenomegaly, and thrombocytosis at diagnosis seem to be independent risk factors for relapse occurrence. When present, monitoring the dog for relapse should be intensified to assess whether prolonged treatment is necessary to prevent relapse.

## Disclosure

Authors declare no off‐label use of antimicrobials.

## Ethics Statement

Authors declare no institutional animal care and use committee or other approval was needed. Authors declare human ethics approval was not needed.

## Conflicts of Interest

Diane Pichard residency received financial support from Royal Canin and Boehringer Ingelheim. The other authors declare no conflicts of interest.
